# Novel insights into STIM1's role in store-operated calcium entry and its implications for T-cell mediated inflammation in trigeminal neuralgia

**DOI:** 10.3389/fnmol.2024.1391189

**Published:** 2024-06-19

**Authors:** Guangyu Cheng, Yu Zhao, Fujia Sun, Qi Zhang

**Affiliations:** ^1^Translational Medicine Research Center of Traditional Chinese Medicine, First Affiliated Hospital of Heilongjiang University of Chinese Medicine, Harbin, China; ^2^Department of Acupuncture, First Affiliated Hospital of Heilongjiang University of Chinese Medicine, Harbin, China; ^3^Preventive Treatment Center, First Affiliated Hospital of Heilongjiang University of Chinese Medicine, Harbin, China

**Keywords:** STIM1, store-operated calcium entry, T lymphocytes, inflammatory cytokines, trigeminal neuralgia, molecular mechanism, bioinformatic analysis, SOCE pathway

## Abstract

This investigation aims to elucidate the novel role of Stromal Interaction Molecule 1 (STIM1) in modulating store-operated calcium entry (SOCE) and its subsequent impact on inflammatory cytokine release in T lymphocytes, thereby advancing our understanding of trigeminal neuralgia (TN) pathogenesis. Employing the Gene Expression Omnibus (GEO) database, we extracted microarray data pertinent to TN to identify differentially expressed genes (DEGs). A subsequent comparison with SOCE-related genes from the Genecards database helped pinpoint potential target genes. The STRING database facilitated protein-protein interaction (PPI) analysis to spotlight STIM1 as a gene of interest in TN. Through histological staining, transmission electron microscopy (TEM), and behavioral assessments, we probed STIM1's pathological effects on TN in rat models. Additionally, we examined STIM1's influence on the SOCE pathway in trigeminal ganglion cells using techniques like calcium content measurement, patch clamp electrophysiology, and STIM1- ORAI1 co-localization studies. Changes in the expression of inflammatory markers (TNF-α, IL-1β, IL-6) in T cells were quantified using Western blot (WB) and enzyme-linked immunosorbent assay (ELISA) *in vitro*, while immunohistochemistry and flow cytometry were applied *in vivo* to assess these cytokines and T cell count alterations. Our bioinformatic approach highlighted STIM1's significant overexpression in TN patients, underscoring its pivotal role in TN's etiology and progression. Experimental findings from both *in vitro* and *in vivo* studies corroborated STIM1's regulatory influence on the SOCE pathway. Furthermore, STIM1 was shown to mediate SOCE-induced inflammatory cytokine release in T lymphocytes, a critical factor in TN development. Supportive evidence from histological, ultrastructural, and behavioral analyses reinforced the link between STIM1-mediated SOCE and T lymphocyte-driven inflammation in TN pathogenesis. This study presents novel evidence that STIM1 is a key regulator of SOCE and inflammatory cytokine release in T lymphocytes, contributing significantly to the pathogenesis of trigeminal neuralgia. Our findings not only deepen the understanding of TN's molecular underpinnings but also potentially open new avenues for targeted therapeutic strategies.

## Introduction

Trigeminal neuralgia (TN) is a severe neurological disorder characterized by the patient experiencing intense facial pain, often described as “electric shocks” or “stabbing sensations,” which impact the patient's quality of life (Cruccu et al., 2016; Di Stefano et al., 2019; Alwardian et al., 2021; Lambru et al., 2022). However, the exact pathogenesis of the disease is still unclear (Huang and Brown, 2022). Existing pain treatment methods, such as medication therapy and surgery, still have limitations in terms of effectiveness, side effects, and potential for recurrence (Hylands-White et al., 2017; Wittkopf and Johnson, 2017; Suresh et al., 2018; Cohen et al., 2021). Therefore, searching for new targets and understanding their roles in TN is to improve therapeutic strategies for TN.

In recent years, the role of calcium signaling in cellular functional regulation has gradually gained attention (Bravo-Sagua et al., 2017; Creamer, 2020; Patergnani et al., 2020; Sukumaran et al., 2021). Store-operated calcium entry (SOCE) is a calcium influx mechanism that regulates intracellular calcium levels, influencing cellular physiological and pathological functions (Chaudhari et al., 2021; Kutschat et al., 2021; Letizia et al., 2022). Stromal Interaction Molecule 1 (STIM1), as an endoplasmic reticulum calcium sensor, could sense changes in endoplasmic reticulum calcium concentration. When the endoplasmic reticulum calcium concentration decreases, STIM1 migrates to the plasma membrane and forms a complex with ORAI1 on the plasma membrane, thereby activating store-operated calcium entry (SOCE) (Yu and Machaca, 2022). However, the specific role of STIM1 in store-operated calcium entry (SOCE) and its function in tumor necrosis remains unclear (Zhao et al., 2020).

T lymphocytes play a crucial role in the immune response by releasing inflammatory factors such as tumor necrosis factor-alpha (TNF-α), interleukin-1β (IL-1β), and interleukin-6 (IL-6), which regulate immune reactions and impact the development of diseases (Geremia et al., 2014; Xu et al., 2018; Moss, 2022). In recent years, the activation of T lymphocytes has been associated with neuropathic pain, including the development of TN. However, further research is still needed to elucidate the specific mechanism (Singh et al., 2022; Dou et al., 2023).

In this study, we conducted bioinformatics analysis and identified that the STIM1 gene might play a crucial role in the occurrence and development of TN. Through comprehensive studies on *in vivo* and *in vitro* experiments, it has been revealed that STIM1 regulates the release of inflammatory factors in T lymphocytes by mediating the store-operated calcium entry (SOCE) pathway, thereby influencing the occurrence of TN. This new finding provides new insights into the pathological mechanism of TN and offers new targets for its treatment.

## Materials and methods

### Bioinformatics analysis

The mouse TN-related chip dataset GSE162284 was obtained through GEO database (https://www.ncbi.nlm.nih.gov/geo/). This dataset included 4 healthy tissue samples and 8 TN tissue samples. Using the “Limma” package in R software, differentially expressed genes (DEGs) were selected between healthy tissue samples and trigeminal neuralgia samples, with thresholds set at |logFC|>0.5 and *P*.adjust < 0.05. The keyword “Store-operated Ca^2+^ entry” was queried on the Genecards website (https://www.genecards.org) to obtain the top 100 genes associated with SOCE. The intersection of these genes was retrieved through the Xiantao Academic website (https://www.xiantao.love/login). Then, a heatmap of the intersected candidate genes was plotted using the “pheatmap” package in R. The filtered genes were imported into the STRING database (https://string-db.org/) for protein interaction analysis. Protein-protein interaction network nodes were sorted by degree value using the “count” package in R. Finally, gene expression analysis was performed using the “ggpubr” package in R (Deng et al., 2020).

### Lentivirus infection

The lentiviral overexpression vector pCDH-CMV-MCS-EF1α-copGFP (oe-, overexpression vector, CD511B-1, System Biosciences, USA) and lentiviral interference vector pSIH1-H1-copGFP (sh-, interference vector, SI501A-1, System Biosciences, USA) were purchased for the development of the lentiviral-based STIM1 overexpression or silencing vector. The silencing sequence is shown in Supplementary Table S1. The lentiviral particles were package into HEK-293T cells (CRL-3216, ATCC, USA) using the lentivirus packaging kit (A35684CN, Invitrogen, USA). After 48 h, the cell supernatant containing lentivirus with a titer of 1 × 10^8^ TU/ml was harvested (Jiang et al., 2017; Belur Nagaraj et al., 2021).

### Constructing a rat model of trigeminal neuralgia

The experiments were conducted using female SD rats weighing ~160–180 g purchased from Beijing Vital River Laboratory Animal Technology Co., Ltd. in Beijing, China. Rats were housed under non-pathogenic conditions at temperatures of 26–28°C and humidity of 50%−65%, with *ad libitum* access to food and water. One week of adaptation feeding should be conducted before the experiment. All animal experiments comply with ethical standards and are approved by Animal Ethics Committee of First Affiliated Hospital of Heilongjiang University of Chinese Medicine (approved number: 2023122950).

The animals were divided randomly into the following groups: Sham group, TN group, TN + sh-NC group, TN + sh-STIM1 group, TN + oe-NC group, TN + oe-STIM1 group, and TN + oe-STIM1 + YM58483 group, with six animals in each group.

On the day before the surgery, the SD rats were placed in the correct lateral position and injected with 5 × 10^6^ U of overexpressed or silenced STIM1 lentivirus (or-STIM1, sh-STIM1) according to the requirements of the groups, serving as the hostile control groups (oe-NC, sh-NC). The injection was performed on the trigeminal ganglion, located 1 mm below the infraorbital foramen on the ventral side of the zygomatic bone, using a 25-gauge needle connected to a neurostimulator (0.15 mA). The injector was removed after 10 min.

The rats were anesthetized by intraperitoneal injection of 3.5% chloral hydrate (1 ml/100 g). After the anesthesia took effect, the rats were placed on the surgical table, and the left facial area for surgery was exposed. After disinfecting the skin of the surgical area, a 5–6 mm incision was made ~8 mm from the lower margin of the nose on the left side of the rat's Whisker pad area. Then, a sterile blunt instrument was used to separate and expose a portion of the infraorbital nerve. Two sutures were placed on the nerve using 5-0 sutures, with a recommended tension that causes slight deformation. The distance between the two sutures was ~2 mm. Finally, use a 3-0 suture to close the skin.

The rats in the sham surgery group were subjected to the same procedural steps, but no infraorbital nerve ligation was performed. On the 14th day after surgery, daily oral administration of YM58483 at 1 mg/kg was performed using HY-100831 (MedChem Express, New Jersey, USA) (Yoshino et al., 2007; Li et al., 2019, 2021; Mehrotra et al., 2019).

### Primary T cell culture

Rat blood collection: Healthy SD rats meeting the criteria were selected, and peripheral blood samples were collected. The collected peripheral blood sample was placed into an anticoagulant collection tube, mixed thoroughly, then centrifuged using a centrifuge machine to separate the supernatant (plasma) from the intermediate white blood cell layer. The separated white blood cells were washed once with PBS and the supernatant was removed by centrifugation. T-cells were isolated using T-cell separation reagent (HY-001067, Huayasi Life Science) for magnetic bead separation of T cells; following the manufacturer's instructions for antibody and bead binding and washing steps. Finally, T cells were isolated and fixed using a PBS solution containing 3.7% formaldehyde after separating them with a separation kit. The fixed cells were then permeabilized with a 0.1% Triton X-100 solution. T cells were labeled with CD3 antibody (14-0030-82, 1:500, ThermoFisher, USA). The labeled cell samples were injected into a flow cytometer, and the fluorescence intensity of the cells was measured by laser excitation. The purity of the T cells obtained was above 90%. The isolated T cells were added to RPMI1640 medium (11875176, ThermoFisher, USA), supplemented with 10% fetal bovine serum (A5669701, ThermoFisher, USA) and 1% antibiotics. The cells were transferred to a culture dish pre-coated with Poly-L-lysine and cultivate T cells in a CO_2_ incubator at 37°C and 5% CO_2_ (Raulf, 2019).

### Acute dissociation of trigeminal ganglion cells

After SD rats were completely anesthetized by inhaling isoflurane at a concentration of 0.6 ml/min, the skull was opened, and a pair of trigeminal ganglia were quickly removed and placed into ice-cold DMEM/F12 medium (A4192002, ThermoFisher, USA). The trigeminal ganglion was transferred to the super clean platform, and micro forceps were used to peel off the superficial fascia of the nerve. The tissue was cut into chunks as much as possible with ophthalmic scissors, transferred the tissue to the digestive fluid, and placed it in a culture dish for complete digestion. The trigeminal nerve tissue was transferred into a 5 ml glass centrifuge tube and gently blown 15 times with a dropper to disperse the tissue. It was allowed to stand for 1 min, add 0.3 ml of culture medium was added, and then gently blown with a 200 μl liquid transfer pipette for 3 min. After letting it stand for 1 min, the suspension of trigeminal ganglion neurons was aspirated after the tissue had settled. They were sown on a 35 mm disposable culture dish coated with 0.01% polylysine, and 0.3 ml of cell culture medium was added. The above steps were repeated until the tissue disappears. The culture dish was placed in a CO_2_ incubator with 5% CO_2_ and 95% O_2_ at 37°C. Subsequently, it was used to detect the regulatory role of STIM1 in SOCE in rat trigeminal ganglion cells, measure Ca^2+^ levels, and perform patch-clamp electrophysiology experiments (Li et al., 2021).

### Flow cytometry sorting of CD3 T lymphocytes from the trigeminal ganglion

The suspension of trigeminal nerve cells was centrifuged in a centrifuge tube at a speed of 1,000 rpm for 5 min. The upper liquid was poured off, and the sediment cells were retained. Cell culture medium was added, and the remaining tissue cell blocks were ground using a tissue homogenizer—repeated washing was performed. Approximately 1 ml of wash buffer was added, and the screening was carried out. The cell suspension was then placed into a column containing CD3-labeled antibody-coated magnetic beads. The cell suspension in the column tube was gently agitated to allow the cells to bind with the CD3 (14-0030-82, 1:50, ThermoFisher, USA) antibody. By adverse selection, non-T cells were removed, leaving T cells. The cells were rinsed with detergent to remove unbound cells and magnetic beads. The cell suspension was transferred into a clean, sterile culture plate, and the T cells were examined using a microscope. Attention was paid to check the integrity and viability of the cells. Target cell samples were collected and isolated, T cells were sorted using flow cytometry, immunofluorescent staining was used to determine the purity of T cells, and the sorted T cells were cultured in a 12-well plate with a coverslip. The experiment was started when the cells reached ~80% confluence. Excess water was removed after quick rinsing with PBS. The cells were immersed in a 2%−4% formaldehyde solution, ensuring complete 2–3 mm submersion. This state was maintained at room temperature for 15 min.

The fixative was removed, and the cells were washed three times with PBS for 5 min each and then incubated in sealing fluid for 60 min. After the sealing liquid was removed, the water droplets around the slide were dried, and diluted primary antibody CD3 (14-0030-82, 1:50, ThermoFisher, USA) was added and incubated overnight at 4°C. Subsequently, the samples were quickly washed three times with PBS for 5 min each. Goat anti-mouse IgG (ab150113, 1:500, Abcam, UK) was incubated under light protection for 1–2 h. Following incubation, it was washed three times with PBS for 5 min each. The purity of the separated T-cells, observed under the microscope, was found to be above 95% (Raulf, 2019; Li et al., 2021).

### H&E staining

The trigeminal ganglion that had been sectioned was stained using the Hematoxylin and Eosin (H&E) staining kit, following the instructions provided by Shanghai Bogu Biotech Co., Ltd. (Shanghai, China). The main steps were as follows: firstly, it was stained with hematoxylin for 10 min at room temperature, then rinsed with running water for 30–60 s; next, it was differentiated using 1% hydrochloric acid alcohol for 30 s, followed by another rinse with running water and soaking for 5 min; then, it was stained with eosin for 1 min; afterward, it underwent gradual dehydration with alcohol (concentrations of 70%, 80%, 90%, 95%, 100%) for 1 min each; subsequently, it was soaked in xylene saturated with stone coal for 1 min, followed by two rounds of transparency treatment in xylene I and II, for 1 min each; finally, the slides were mounted in neutral mounting medium in a ventilated hood, and the morphological changes of each group were observed and captured using an optical microscope (BX50; Olympus Corp, Tokyo, Japan) (Jing et al., 2021; Mao et al., 2022).

### Immunohistochemical staining

After embedding and slicing the trigeminal ganglia of each group of rats, they were baked at 60°C for 20 min. Subsequently, they were soaked in a xylene solution for 15 min, followed by immersion in absolute alcohol for 5 min, with a change of absolute alcohol and an additional 5 min of soaking. Following this, the sections were sequentially hydrated in 95 and 70% alcohol, with each alcohol being hydrated for 10 min. Each section was treated with 3% H_2_O_2_ and incubated at room temperature for 10 min to inhibit endogenous peroxidase activity. The sections were then treated with a citric acid buffer and steamed in the microwave for 3 min, followed by the addition of antigen retrieval solution, which was left at room temperature for 10 min before being washed three times with PBS. They were then incubated with a normal goat serum blocking solution (E510009, Shanghai Bioengineering Co., Ltd.) at room temperature for 20 min and subsequently incubated with the primary antibody CD3 (14-0030-82, 1:100, ThermoFisher, USA) overnight at 4°C. After three washes with PBS, the sections were incubated with a goat anti-mouse IgG secondary antibody (ab150113, 1:500, Abcam, UK) for 30 min. Following PBS washing, the DAB chromogenic reagent kit (P0203, Biotech, Shanghai, China) was applied, and one drop of color agents A, B, and C was added to the specimen. The specimen was incubated for 6 min for color development. It was then stained with hematoxylin for 30 s, sequentially dehydrated in 70%, 80%, 90%, and 95% ethanol, and finally in absolute ethanol for 2 min each. The sections were soaked twice in xylene for 5 min and sealed with neutral resin. The slide was observed under the Olympus BX63 upright microscope (Li et al., 2022).

### Transmission electron microscopy

Euthanasia was performed on rats under deep anesthesia, followed by the chest being opened to expose the heart and aorta. The needle was carefully inserted into the left ventricle, secured at the origin of the aorta, and the right atrium was flushed with 0.9% saline solution until the saline in the right atrium cleared. The tissue was infused with 4% formaldehyde overnight. Subsequently, the intact trigeminal ganglion was fixed in 4% polyformaldehyde overnight. Long tissue sections with a volume of 1 mm3 were then obtained from the terminal end of the trigeminal ganglion and soaked in 1% O_S_O_4_ for 1 h and uranyl acetate for 2 h. Following this, gradient dehydration was carried out using ethanol and epoxypropane, and the tissue was embedded with epoxy resin. Ultra-thin tissue sections were stained with uranyl acetate. The tissue was observed and captured using the Hitachi H-7650 transmission electron microscope (Hitachi, Tokyo, Japan) (Yang et al., 2022).

### Rat orofacial mechanical pain threshold testing

In this experiment, the mechanical pain threshold of the oral surface of rats was measured using von Frey filaments. Each time the right buccal area was stimulated, von Frey filaments were applied with a stimulus interval of 10 s. The bending of the bristle and the intensity of the bristle were recorded during observation of the oral facial pain response. Baseline data was obtained by recording the mechanical pain threshold of each group of rats the day before the surgery. On the 3rd, 5th, 7th, 9th, 11th, 13th, and 14th day after surgery, the mechanical pain threshold of each group of rats was measured again on the second day (i.e., the 15th day after surgery) following treatment with YM58483 to evaluate their mechanical pain threshold (Li et al., 2021).

### The latency period of the hot foot reflex is shortened

Prior to the surgery, the rats underwent adaptive training. For postoperative testing, rats with calm responses to training stimuli and intact oral, facial hair, and skin were selected. The facial whisker region of the rats was exposed to a high-intensity beam of radiation, and observations were made to determine whether they exhibited rapid head shaking or blinking as avoidance responses. The time from the onset of high-intensity radiation to the manifestation of avoidance responses by the rats was defined as the latency period of thermal stimulation response. A thermos with a temperature of 50°C was used, and measurements were taken at a distance of 8 centimeters from the oral and facial test areas during the experiment (Li et al., 2021).

### Spontaneous asymmetric facial modifications

The rats were individually housed in transparent acrylic observation cages. Once the rats had acclimated to their environment, the number of times they engaged in facial asymmetrical self-grooming autonomously within 5 min was observed. In a quiet setting, the rats' faces were continuously stimulated by the experimenters. If a rat exhibited the behavior of grooming and scratching the stimulated area more than three times, it was deemed to be spontaneously scratching its face asymmetrically. Behavior assessment utilized a scoring system, assigning a score of 1 to each instance of spontaneous asymmetrical scratching behavior. The testing was conducted on the 14th day after the surgery and the 15th day after the administration of YM58483 (Li et al., 2021).

### Determination of Ca^2+^ content

Cells were labeled with 2 mM Fura-2-AM (Life Technologies; Catalog number F1201) in the growth medium for 30 min. Following this, the cells were connected to a 96-well imaging plate coated with 0.01% poly-L-lysine (w/v; HY-126437A, MedChem Express, New Jersey, USA) for 10 min and washed twice with Ca^2+^-free Ringer's solution (155 mM NaCl, 4.5 mM KCl, 2 mM CaCl_2_, 1 mM MgCl_2_, 10 mM D-glucose, and 5 mM Na-HEPES). To deplete Ca^2+^ stores within the cells, they were treated with 1 mM thapsigargin (TG; #112758, Cell Signaling Technology, Danvers, MA, USA) in Ca^2+^-free Ringer's solution. In order to induce SOCE, 1 mM Ca^2+^ was added to the Ringer's solution. Changes in the intracellular Ca^2+^ concentration were analyzed using the FlexStation 3 multifunctional microplate reader (Molecular Devices), with excitation wavelengths of 340 and 380 nm, respectively (Secondo et al., 2019).

### Whole-cell patch clamp recording

Single-cell ICRAC (CRAC current) was recorded using the whole-cell patch clamp technique at room temperature. The external bath solution contains 145 mM NaCl, 5 mM KCl, 1 mM CaCl2, 1 mM MgCl_2_, 10 mM HEPES, and 10 mM glucose (pH 7.4), while the pipette solution contains 145 mM CsCl, 8 mM NaCl, 10 mM MgCl_2_, 10 mM HEPES, 10 mM EGTA, and 2 mM Mg-ATP (pH 7.2). ICRAC and store-operated calcium currents were recorded with a 100-ms voltage waveform (ranging from +90 to −120 mV), with recordings taken every 10 s from a holding potential of −15 mV. The magnitude of ICRAC is measured at −120 mV. ICRAC was normalized to membrane capacitance to account for potential changes in cell size caused by specific manipulations (measured in pA/pF) (Secondo et al., 2019).

### Immunofluorescence staining

Trigeminal ganglion cells were seeded onto 5 × 5 mm coverslips in a six-well plate, with 5 × 10^4^ cells in each well. The cells were fixed in PBS containing 4% paraformaldehyde for 20 min. Subsequently, the cells were permeabilized with 0.1% Triton X-100 in PBS for 30 min and then blocked with 5% bovine serum albumin at room temperature for 1 h. Next, overnight incubation at 4°C was performed using rabbit STIM1 (PA5-85387, 1:500, ThermoFisher, USA) and mouse ORAI1 (SAB3500126, 1:500, Sigma-Aldrich, USA) antibodies. Subsequently, secondary antibodies were added, including Goat anti-rabbit IgG (Alexa Fluor 488; 1:1,000, ab150077, Abcam, Cambridge, UK) and Goat anti-mouse IgG (Alexa Fluor 594; 1:1,000, ab150116, Abcam, Cambridge, UK). The samples were incubated at room temperature, avoiding light, for 1 h. DAPI (D9542, Sigma-Aldrich, USA) was used to label cell nuclei at room temperature with a labeling time of 10 min. Detecting chemical fluorescence using laser scanning confocal microscopy. The Pearson correlation coefficient value is used to indicate the degree of co-localization, divided into three levels: strong (0.49–1.0), moderate (0.1–0.48), and weak (−1 to 0.09) (Li et al., 2018; Ko et al., 2020).

### Live cell confocal microscopy imaging

All fluorescence images were captured using the Olympus FLUOVIEW FV1000 confocal microscope equipped with a 60 × oil immersion objective within 48–72 h after transfection. Time-lapse imaging was performed to observe STIM1-YFP transfected T cells. Then, use a 515 nm laser line to excite YFP and acquire images of YFP emission using a 535–565 nm wavelength, with an interval of 20 s per acquisition. For T cells co-transfected with STIM1-YFP and ORAI1-CFP, excitation was performed using 515 and 458 nm laser lines, and images of YFP and CFP were acquired every 2 min. All experiments were carried out at room temperature using Tyrode solution containing the following components: 140 mM NaCl, 5 mM KCl, 1 mM MgCl_2_, 2 mM CaCl_2_, 10 mM HEPES, and 5.6 mM glucose (pH 7.4). The number of STIM1 puncta was calculated using OLYMPUS FLUOVIEW Ver.3.1b software. The regions of interest could be determined by randomly selecting 10 × 10 μm^2^ square areas within the cell. The number of puncta was counted using the same region before and after TG treatment. The characteristic of the spot is the high fluorescence intensity region with a diameter of 0.4–2.0 μm (Gao et al., 2016).

### Proximity ligation assay (PLA)

The cell fixation and permeabilization were carried out following the same method as that used in the immunofluorescence assay. The rabbit-derived STIM1 antibody (PA5-85387, 1:500, ThermoFisher, USA) and the mouse-derived ORAI1 antibody (SAB3500126, 1:500, Sigma-Aldrich, USA) were each combined with a pair of PLA probes (anti-rabbit DUO92002, DUO92004, Sigma-Aldrich, USA). The probes were connected, signals were amplified, and the samples were stained with DAPI (D9542, Sigma-Aldrich, USA) as per the manufacturer's instructions. Subsequently, representative images were captured using the inverted microscope from Carl Zeiss (Germany, Göttingen). The images were analyzed using ImageJ software to quantify the percentage of spots in each DAPI-positive cell nucleus with PLA signals (Zhu et al., 2021; Yuan et al., 2022).

### Western blot

Total protein was extracted from tissues using RIPA lysis buffer containing PMSF (P0013C, Beyotime, Shanghai, China). After being incubated on ice at 4°C for 30 min, the samples were centrifuged at 8,000 g for 10 min, and the supernatant was collected. The total protein concentration was determined using the BCA assay kit (23227, ThermoFisher, USA). The protein (50 μg) was dissolved in 2 × SDS sample buffer, boiled for 5 min, and then subjected to SDS-PAGE gel electrophoresis, with the proteins being transferred to the PVDF membrane. The membrane was then incubated with diluted primary antibodies, including mouse IL-6 (ARC0962, 1:50, ThermoFisher, USA), rabbit IL-1β (ab254360, 1:1,000, Abcam, Cambridge), rabbit TNF-α (ab205587, 1:1,000, Abcam, Cambridge, UK), rabbit STIM1 (PA5-85387, 1:500, ThermoFisher, USA), mouse ORAI1 (ab175040, 1:1,000, Abcam, Cambridge, UK), and rabbit GAPDH (ab181602, 1:10,000, Abcam, Cambridge, UK) as references, after being incubated with 5% skim milk powder to seal the membrane at room temperature for 1 h. Subsequently, the membrane was incubated overnight at 4°C, washed thrice with TBST for 10 min each time, and then incubated with the secondary antibodies, anti-goat IgG H&L (HRP) conjugated with HRP (ab97051, 1:2,000, Abcam, Cambridge, UK) and anti-mouse IgG H&L (HRP) conjugated with HRP (ab6728, 1:2,000, Abcam, Cambridge, UK) for 1 h. Equal amounts of Solution A (peroxidase luminescent substrate containing accessory reagents and diluents for dissolving and diluting protein probes) and Solution B (enhancer, luminescent substrate, and enhancer for reacting with probes and generating a fluorescent signal) from the ECL fluorescent detection kit [abs920, Applied Biological Sciences (Shanghai) Co., Ltd., Shanghai, China] were mixed, and then added dropwise onto the membrane. Images were captured using the Bio-Rad imaging system (BIO-RAD Corporation, USA) and analyzed using Quantity One v4.6.2 software. The relative protein content was calculated as the ratio of the grayscale values of the protein bands of interest to the grayscale value of the GAPDH protein band. The experiment was repeated three times, and the average value was taken (Jiang et al., 2006).

### ELISA

The cells were first placed in a calcium-free Tyrode solution composed of 140 mM NaCl, 5 mM KCl, 1 mM MgCl_2_, 10 mM HEPES, and 5.6 mM glucose and incubated for 2 min. Subsequently, the cells were treated with 1 μM TG for 5 min. Following this, the calcium-free Tyrode solution was replaced with a medium containing TG, and the cells were cultured for 24 h. Finally, the cells were centrifuged at 1,000 rpm for 5 min, and following the manufacturer's instructions, an ELISA assay kit was used to measure the levels of pro-inflammatory cytokines, including TNF-α, IL-6, and IL-1β, secreted in the supernatant collected from primary T cells derived from rats. The ELISA kits utilized in the experiment were as follows: Rat IL-6 (PI328, Beyotime, China), Rat IL-1β (PI303, Beyotime, China), and Rat TNF-α (PT516, Beyotime, China) (Gao et al., 2016; Chen et al., 2021).

### Detection of proportions of various T lymphocyte subsets using flow cytometry

Each group of test cells was collected into clean 15 ml centrifuge tubes. They were washed twice with pre-cooled PBS, centrifuged at 350 × g for 5 min, and the supernatant was discarded. The cell pellet was then resuspended in cell staining buffer (MX1504-100ML, MKbio, Shanghai Maokang Biological Technology Co., Ltd.) and the cell suspension concentration was adjusted to 1 × 10^7^ cells per milliliter. Subsequently, 100 μl of cell suspension per tube was transferred into a 5 ml flow tube. To each tube, 5 μl of CD3 antibody specific for T lymphocytes (14-0030-82, 1:50, ThermoFisher, USA), IL-6 (ARC0962, 1:50, ThermoFisher, USA), IL-1β (ab254360, 1:50, Abcam, Cambridge, UK), and TNF-α (ab205587, 1:100, Abcam, Cambridge, UK) were added. The tubes were mixed well and incubated at room temperature, protected from light, for 15–20 min. Following this, 2 ml of cell staining buffer was added to each tube, then centrifuged at 350 × g for 5 min, the supernatant was discarded, and 200 μl of cell staining buffer was added to each tube. The mixtures were well combined, and a flow cytometer was used for detection within 1 h. Three replicate experiments with three duplicate wells in each group were performed. The experimental results were analyzed using FlowjovX.7, which is a professional flow cytometry analysis software. The peaks were displayed using scale gates or scatter plots with a “cross” gate in the four quadrants to show the proportion (%) of each cell subset in the total population (Weidinger et al., 2013).

### CCK-8

The CCK-8 kit (CA1210, Beijing Solaibao Technology Co., Ltd., Beijing, China) was utilized for conducting cell proliferation experiments, and the results were statistically analyzed. Cells in the logarithmic growth phase were harvested and pre-cultured in a 96-well plate with 1 × 10^4^ cells per well for 24 h. Subsequently, cell transfection was performed based on groups, and 10 μl of CCK-8 reagent was added within 48 h post-transfection. Following this, the absorbance values at a wavelength of 450 nm were measured using an enzyme immunoassay reader to assess the proliferation of cells in the culture medium. The relative absorbance values of these samples were positively correlated with cell proliferation. A bar graph of cell viability for each group was then generated (Huang et al., 2018).

### Statistical analysis

All data were analyzed using SPSS 22.0 statistical software (SPSS, Inc., Chicago, IL, USA) and GraphPad Prism 9.5. Measurement data is expressed in the form of mean ± standard deviation (Mean ± SD). The non-paired *t*-test is used to compare the differences between two groups, and a one-way analysis of variance is used to compare differences among multiple groups. The homogeneity of variance was tested using Levene's test. Dunnett's *t*-test and LSD-*t*-test were used for pairwise comparisons if the variances were homogenous. If variances are unequal, use Dunnett's T3 test. *P* < 0.05 indicates that the difference in comparison between the two groups is statistically significant.

## Results

### Identification and validation of STIM1 as a key regulator in trigeminal neuralgia pathogenesis: insights from bioinformatics analysis and *in vivo* experiments

**Figure 1:**
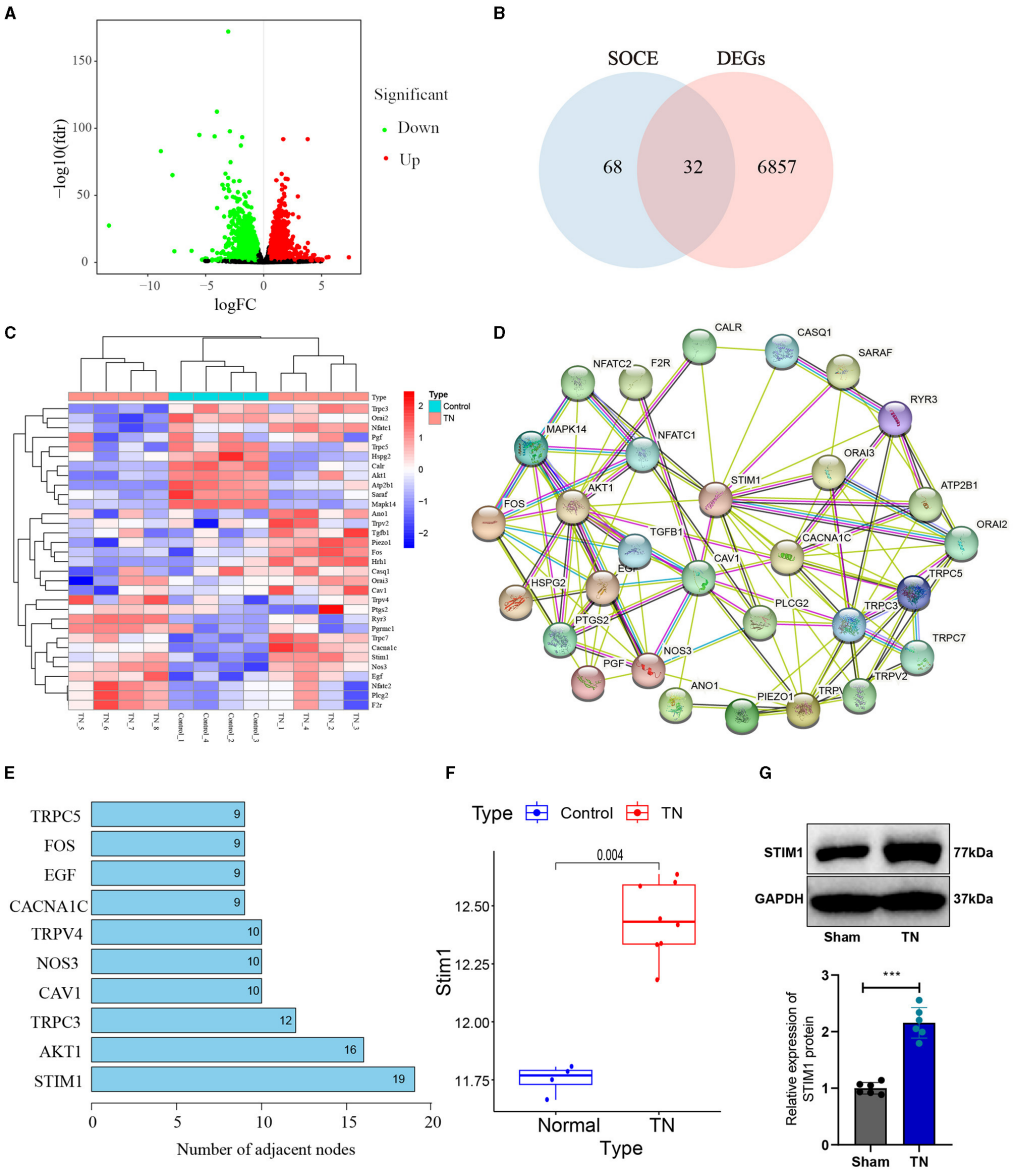
Identification of target gene STIM1 using bioinformatics analysis. **(A)** Volcano plot of differentially expressed genes in the trigeminal neuralgia chip GSE162284 (Control group, *n* = 4; TN group, *n* = 8). The *x*-axis represents the log_10_ *p*-value and the *y*-axis represents logFC. Red dots indicate genes with high expression in the disease, and green dots indicate genes with low expression. **(B)** The intersection of SOCE-related genes and differentially expressed genes in the chip (the middle section represents the intersection of the two datasets). **(C)** Heatmap of differentially expressed SOCE-related proteins (Control group, *n* = 4; TN group, *n* = 8). The *x*-axis represents sample numbers, the *y*-axis represents gene names, the upper dendrogram represents sample clustering, and the histogram in the top-left corner represents the color gradient. **(D)** Protein-protein interaction network of candidate genes encoding proteins in the String database. **(E)** Top 10 proteins displaying the degree of protein interaction encoded by the intersected candidate genes. **(F)** Protein expression statistics of STIM1 in the GSE12470 dataset (Control group, *n* = 4; TN group, *n* = 8). **(G)** Western blot detection of STIM1 expression levels in the trigeminal nerve of Sham group and TN group rats (Control group, *n* = 6; TN group, *n* = 6). ***P < 0.001.

Trigeminal neuralgia is a common neurological disorder characterized by paroxysmal severe pain in the facial distribution of the trigeminal nerve. Treating trigeminal neuralgia is still a huge challenge (Liu et al., 2015). To investigate the possible molecular mechanisms underlying trigeminal neuralgia, we obtained the differentially expressed genes (DEGs) from the trigeminal neuralgia microarray GSE162284 in the GEO database (Figure 1A). The calcium signaling pathway is closely associated with the development of trigeminal neuralgia (Gualdani et al., 2021). SOCE regulates chronic pain in the dorsal root ganglia and spinal cord (Zhang and Hu, 2020). Therefore, we further performed an intersection analysis between these differentially expressed genes and the top 100 genes ranked by relevance scores in the gene cards database related to SOCE (Figure 1B) and identified 32 differentially expressed SOCE-related genes in trigeminal neuralgia (Figure 1C). We will then input the proteins corresponding to these 32 candidate genes into the String database to obtain their protein-protein interaction relationships (Figure 1D). Display the number of target proteins for each protein based on the Degree values (Figure 1E), with STIM1 having the highest number of target proteins and exhibiting high expression in the GSE162284 dataset (Figure 1F). Therefore, we selected STIM1 as the target gene for subsequent experimental validation. We used SD rats and constructed a trigeminal neuralgia (TN) model using infraorbital nerve chronic constriction injury (IoN-CCI). WB results showed (Figure 1G): the expression of STIM1 in the TN group was upregulated compared to the Sham group.

In summary, our results indicate that the upregulation of STIM1 expression may play an essential role in the pathogenesis and progression of trigeminal neuralgia.

### STIM1 knockdown alleviates tissue structural damage and pain behavior in trigeminal neuralgia-induced rats

**Figure 2:**
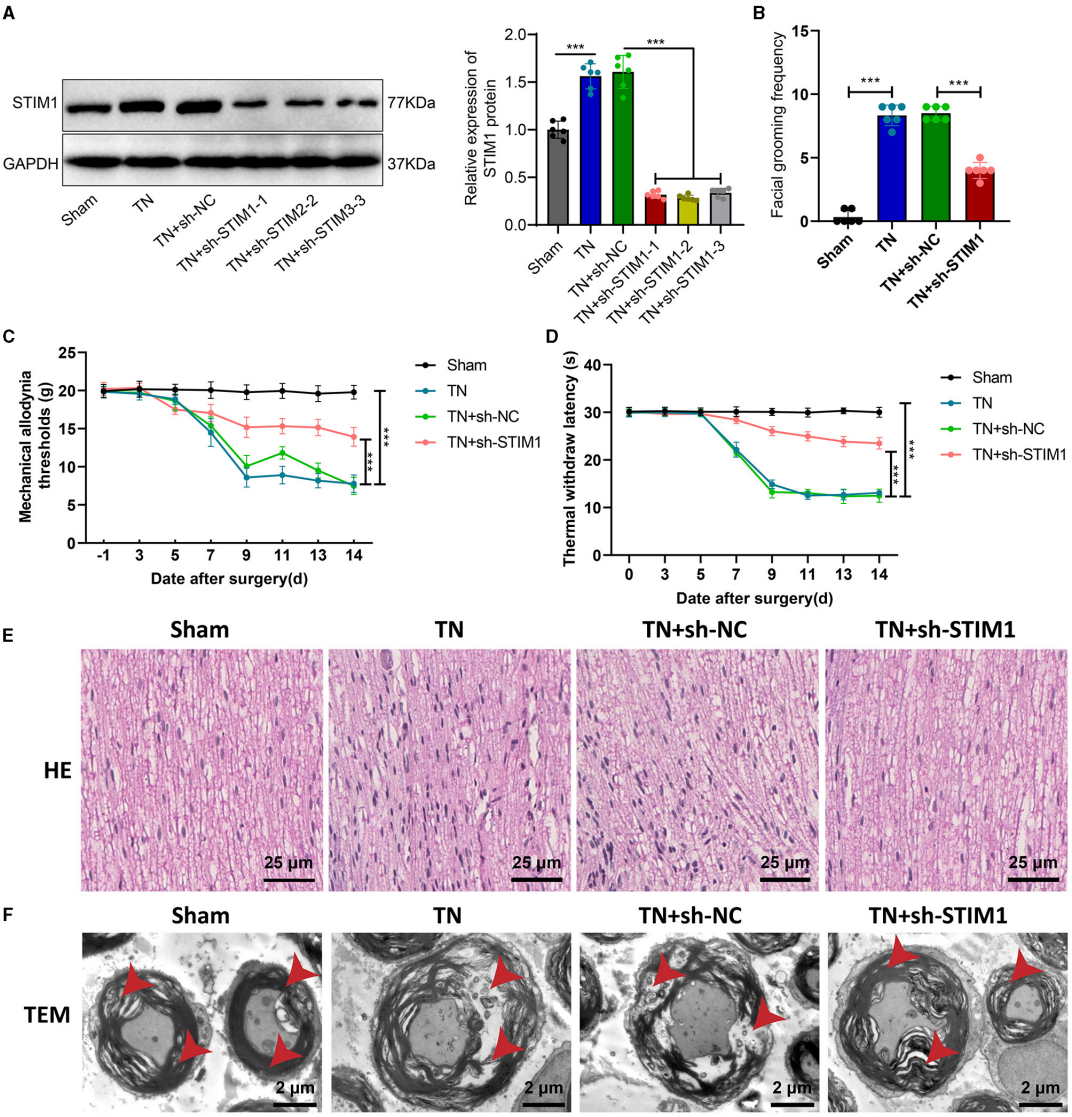
Effect of STIM1 knockdown on trigeminal neuralgia. **(A)** Western blot detection of STIM1 knockdown efficiency in TN group rats, six rats per group. **(B)** Spontaneous asymmetrical facial grooming experiment was used to assess grooming behavior in each group of rats, six rats per group. **(C)** Hot plate reflex latency test to detect changes in thermal pain threshold in each group of rats, six rats per group. **(D)** Von Frey experiments to detect changes in mechanical pain threshold in each group of rats, six rats per group. **(E)** HE staining to observe pathological changes in the trigeminal ganglion of each group of rats ( × 400), six rats per group. Blue represents the neuronal cell nuclei of the trigeminal ganglion, while red represents other types of cellular structures or tissue components within the trigeminal ganglion. **(F)** Microscopic structural changes in the trigeminal ganglion of each group of rats ( × 5,000), six rats per group. Ns indicates no difference between the two groups (*P* > 0.05), *** indicates a highly difference (*P* < 0.001).

The influence of organizational structure and pain behavior. We performed STIM1 knockdown in TN rats. The WB results show (Figure 2A): after silencing STIM1, the expression of STIM1 decreased. Among them, the third group showed the best silencing effect. Therefore, we chose that group for subsequent experiments.

Facial spontaneous grooming behavior was observed and quantified in TN-treated rats compared to the Sham group, as indicated by the results of facial self-grooming detection (Figure 2B). However, after silencing STIM1, spontaneous facial grooming behavior in rats was reduced. Mechanical pain threshold and thermal pain threshold detection results (Figures 2C, D) showed that compared with the Sham group, the mechanical pain threshold and thermal pain threshold of rats in the TN group were reduced on the right side of the face. However, following the inhibition of STIM1, the mechanical pain threshold and thermal pain threshold in the right face of the rats were increased.

The HE staining results of rat samples (Figure 2E) showed that the nerve fibers and Schwann cells were arranged neatly in the Sham group, with regular cell morphology and clear structure. The arrangement of nerve fibers in the TN group appears slightly disordered, with the proliferation of Schwann cells, axonal swelling, infiltration of lymphocytes, and changes in the myelin sheath. Compared with the TN group, the TN + sh-STIM1 group showed an orderly arrangement of nerve fibers, increased improvement in nerve fiber degeneration, and reduced inflammatory response.

Electron microscopy results (Figure 2F) showed that the trigeminal ganglion nerve fibers in the Sham group rats had regular axonal morphology, with uniform and transparent myelin sheath layers, consistent thickness, and circular or oval shape. Circular mitochondria were visible within the cell cytoplasm. Compared to the control group, the cross-sectional area of the trigeminal ganglion nerve fibers in the TN group rats increased, with thicker myelin sheaths, loose lamellar plates, disrupted structure, and localized myelin sheath fractures. However, after silencing STIM1, the tissue structure improved.

The above experimental results indicate that silencing STIM1 could alleviate the effects of tissue structure and pain behavior caused by trigeminal neuralgia.

### STIM1 modulates SOCE and inflammatory cytokine release in T lymphocytes: insights into its role in trigeminal neuralgia pathogenesis

Neuropathic pain involves not only neuronal phenomena but also immune responses. Hypertrophic cells, granulocytes, macrophages, and T lymphocytes infiltrate damaged surrounding nerves. These cells promote neuropathic pain by secreting inflammatory mediators such as pro-inflammatory cytokines and chemokines (Machelska, 2011). Regulating intracellular calcium ion concentration is an important signaling mechanism for regulating the function of immune cells (Weidinger et al., 2013). SOCE is involved in regulating the release of inflammatory factors by T lymphocytes. After T lymphocytes receive stimulation signals, SOCE will be activated, increasing intracellular calcium ion concentration. This process will trigger T lymphocytes to release inflammatory factors, such as interleukin-2 and TNF-α (Vaeth et al., 2020). STIM1 is a necessary protein for implementing store-operated calcium entry (SOCE) in T cells. Loss-of-function or mutation in the STIM1 gene eliminates Ca^2+^ influx in T cells, leading to patient immunodeficiency. Therefore, we further investigated whether STIM1 could regulate trigeminal neuralgia by modulating the release of inflammatory cytokines in T lymphocytes.

**Figure 3:**
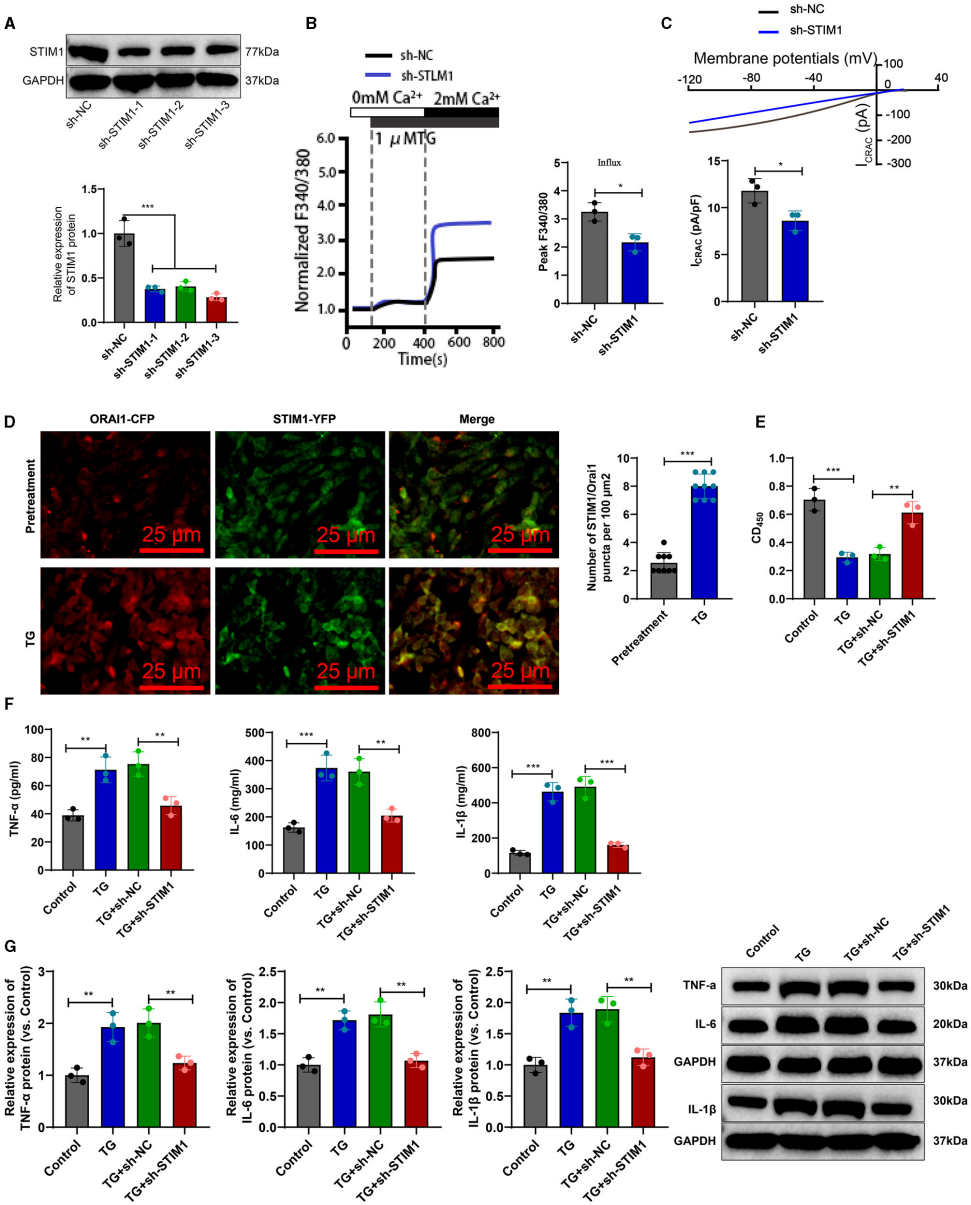
STIM1 knockdown mediates inhibition of SOCE and T cell inflammatory cytokine release. **(A)** Western blot detection of STIM1 knockdown efficiency after STIM1 knockdown, all cell experiments repeated three times. **(B)** Determination of Ca^2+^ content in T cells of each group, all cell experiments repeated three times. **(C)** Patch clamp technique for electrophysiological detection of whole-cell records and quantification of ICRAC in each group of T cells, all cell experiments repeated three times. **(D)** Co-localization of STIM1 and ORAI1 in cells observed under confocal microscopy (scale bar = 40 μm). Nine fields of view were taken for each group. **(E)** CCK-8 assay to detect cell viability in each group of T cells, all cell experiments repeated three times. **(F)** Elisa assay to detect expression of inflammatory cytokines TNF-α, IL-6, and IL-1β in each group of T cells, all cell experiments repeated three times. **(G)** Western blot detection of expression of inflammatory cytokines TNF-α, IL-6, and IL-1β in each group of T cells, all cell experiments repeated three times *P < 0.05, **P < 0.01, ***P < 0.001. TG, thapsigargin.

To investigate the effect of STIM1 on the production of inflammatory cytokines in T cells, we silenced STIM1 in primary T cells. The WB results showed (Figure 3A): a decrease in STIM1 expression, with the third silencing effect being the most effective. Therefore, we selected it for further experiments. Firstly, the role of STIM1 in SOCE in T cells was analyzed. The Ca^2+^ content detection results (Figure 3B) showed that, compared with the Control group, silencing STIM1 reduced thapsigargin (TG)-induced SOCE during depletion of endoplasmic reticulum Ca^2+^ stores. The results of the patch clamp technique electrophysiology experiment (Figure 3C) showed that compared to the Control group, the activation of ICRAC mediated by SOCE in T cells was increased, and silencing STIM1 reduced the activation of ICRAC in the trigeminal ganglia. The regulation of SOCE is mainly achieved through the interaction between STIM and ORAI proteins in the endoplasmic reticulum membrane and the cell membrane. When the intracellular calcium ion concentration decreases, STIM1 will aggregate on the surface of the endoplasmic reticulum membrane, interact with ORAI1 on the cell membrane, activate the ORAI1 channel protein, and allow the formation of pore-like structures, thereby allowing extracellular calcium ions to enter the cell and increase the intracellular calcium ion concentration (Courjaret et al., 2023). To examine the co-localization of STIM1 and ORAI1 in T cells, we co-transfected ORAI1-CFP and STIM1-YFP into T cells. Confocal microscopy revealed (Figure 3D) that STIM1-YFP was uniformly expressed throughout the entire cell except for the nucleus in untreated cells. After the addition of TG, the distribution of ORAI1-CFP underwent changes; TG triggered the recombination of STIM1 and ORAI1, leading to a enhancement of the co-localization of STIM1 and ORAI1. It indicates that STIM1 interacts with ORAI1 when Ca^2+^ stores are depleted. This result further supports the view that depletion of endoplasmic reticulum Ca^2+^ storage leads to STIM1 translocation, which activates ORAI1 and subsequently induces calcium influx into T cells.

CCK-8 results showed (Figure 3E): compared with the Control group, cell viability was decreased in the TG group; after silencing STIM1, T-cell activity increased significantly. Elisa assay was used to measure the expression levels of inflammatory factors in T cells from different groups. The results (Figure 3F) showed that compared to the Control group, the expression of IL-6, IL-1β, and TNF-α in T cells from the TG group increased. Silencing STIM1 decreased the expression of IL-6, IL-1β, and TNF-α in T cells. WB analysis was performed to examine the expression of inflammatory factors in T cells from different groups. The results (Figure 3G) showed that the expression of IL-6, IL-1β, and TNF-α in T cells from the TG group increased compared to the Sham group. Silencing STIM1 decreased the expression of IL-6, IL-1β, and TNF-α in T cells. The above results indicate that STIM1 regulates both SOCE and the release of inflammatory factors in T lymphocytes.

### STIM1 regulates SOCE in trigeminal ganglion cells: implications for trigeminal neuralgia mechanisms and potential therapeutic intervention

Ca^2+^ channels play an essential role in the mechanism of pain generation. Voltage-dependent Ca^2+^ channel auxiliary subunit expression increases in the damaged neuronal membranes, leading to membrane depolarization, Ca^2+^ influx, and action potential generation (Kraychete et al., 2008). SOCE participates in a variety of cellular physiological and pathological processes. Electrophysiological studies have shown that the transmembrane transport of calcium ions is closely related to neuronal depolarization, which affects the occurrence and development of pain responses (Kuroda et al., 2013). STIM1 is the leading participant of SOCE (Silva-Rojas et al., 2020). Therefore, we speculate that STIM1 promotes the specific mechanism of trigeminal neuralgia by regulating SOCE.

**Figure 4:**
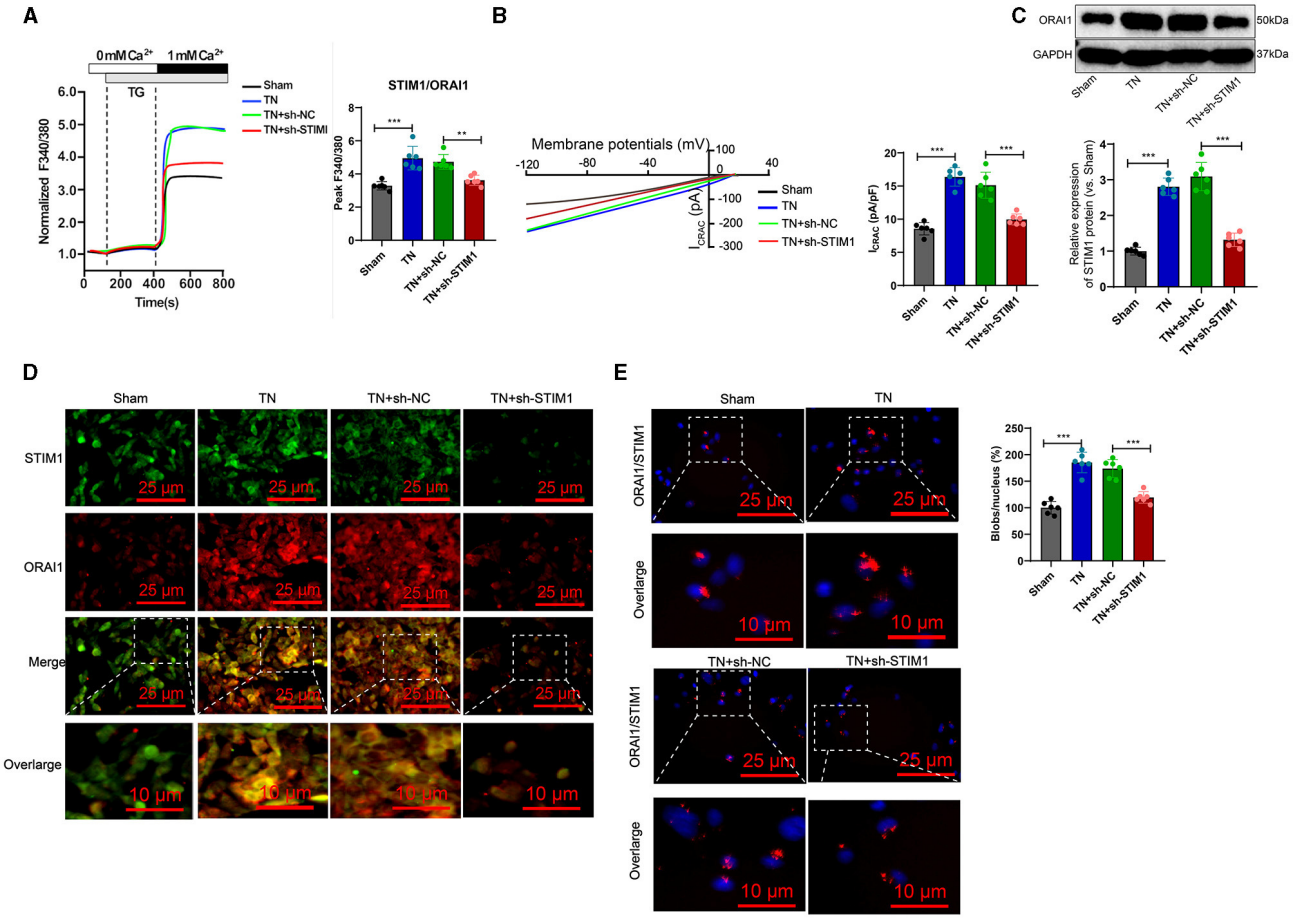
STIM1 knockdown reduces Ca^2+^ levels in trigeminal nerve cells and inhibits SOCE. **(A)** Determination of Ca^2+^ content in trigeminal ganglion cells of each group, six rats per group. **(B)** Patch clamp technique for electrophysiological detection of whole-cell records and quantification of ICRAC in each group of trigeminal ganglion cells, six rats per group. **(C)** Western blot detection of expression levels of ORAI11 in each group of trigeminal ganglion cells, six rats per group. **(D)** Co-localization of STIM1 and ORAI1 in cells observed under confocal microscopy, six rats per group. **(E)** Proximity ligation assay to detect the interaction between STIM1 and ORAI1 in the trigeminal ganglion of each group of rats (scale bar = 25 μm). Six rats per group *P < 0.05, **P < 0.01, and ***P < 0.001. TG, thapsigargin.

The trigeminal ganglion is the gathering area of sensory nerve cell bodies for the trigeminal nerve and is closely associated with trigeminal neuralgia (Kc et al., 2022). Therefore, we analyzed the role of STIM1 in store-operated calcium entry (SOCE) in the trigeminal ganglion of trigeminal neuralgia rats. The Ca^2+^ content detection results (Figure 4A) showed that, when inducing store-operated calcium entry (SOCE) by depleting endoplasmic reticulum Ca^2+^ stores with thapsigargin (TG), the TN group of rats exhibited increased Ca^2+^ flux in trigeminal ganglion cells compared to the Sham group. Silencing STIM1 reduced Ca^2+^ flux in trigeminal ganglion cells by 30%−50%. The electrophysiological experimental results of the membrane clamp technique showed (Figure 4B): compared with the Sham group, the activation of ICRAC mediated by SOCE in the trigeminal ganglion cells of rats in the TN group increased, while silencing STIM1 decreased the activation of ICRAC in the trigeminal ganglion.

Deletion of STIM1 may lead to downregulation of ORAI1 expression in a dependency manner. WB detection shows the production of ORAI1 protein in the trigeminal ganglion. The results (Figure 4C) show that compared to the Sham group, the TN group of rats had an increased amount of ORAI1 protein in the trigeminal ganglion. However, after silencing STIM1, the ORAI1 protein content decreased. Immunofluorescence assay results showed (Figure 4D): compared with the Sham group, the co-localization of STIM1 and ORAI1 in the trigeminal ganglia of TN group rats increased by ~two-fold, while silencing STIM1 reduced the co-localization of STIM1 and ORAI1 in the trigeminal ganglia. The results of the nearby coupling experiments (Figure 4E) demonstrated that compared to the Sham group, the interaction between STIM1 and ORAI1 in the trigeminal ganglia of TN group rats was increased. Silencing STIM1, however, resulted in decreased interaction between STIM1 and ORAI1 in the trigeminal ganglia. This result further supports that STIM1 regulates the SOCE pathway by activating STIM1. In conclusion, silencing STIM1 reduces the Ca^2+^ levels in trigeminal nerve cells and inhibits SOCE.

### STIM1 mediation of SOCE in T cells augments inflammatory cytokine release in trigeminal neuralgia: modulatory effects of YM58483

**Figure 5:**
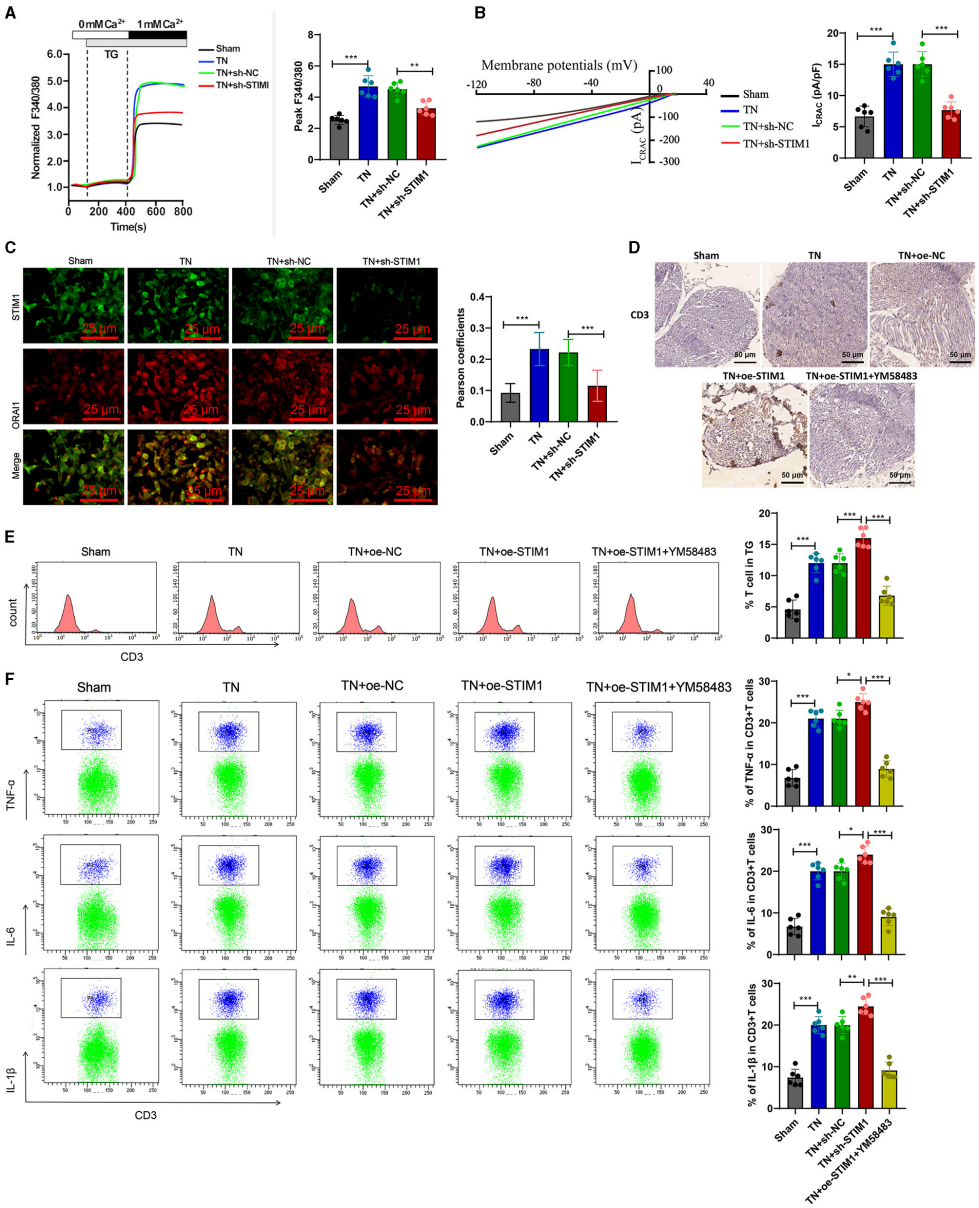
Overexpression of STIM1 promotes the release of inflammatory cytokines in SD rat T cells through mediating SOCE. **(A)** Determination of Ca^2+^ content in T cells of each group, six rats per group. **(B)** Patch clamp technique for electrophysiological detection of whole-cell records and quantification of ICRAC in T cells of each group, six rats per group. **(C)** Co-localization of STIM1 and ORAI1 in T cells was observed under confocal microscopy. The statistical plot shows the Pearson correlation coefficient (arrow indicates the enlarged area, scale bar = 10 μm, each group contains 30 cells), six rats per group. **(D)** Immunohistochemical detection of T cell distribution and expression in the trigeminal ganglion of each group of rats (scale bar = 100 μm), six rats per group. **(E)** Flow cytometry analysis of T cell quantity in the trigeminal ganglion of each group of rats, six rats per group. **(F)** Flow cytometry analysis of inflammatory cytokine release from T cells in the trigeminal ganglion of each group of rats, six rats per group. ***P < 0.001, six rats per group. oe-STIM denotes overexpression of STIM1, while YM58483 refers to a store-operated calcium entry (SOCE) inhibitor. *P < 0.05 and **P < 0.01.

To further validate whether intracellular SOCE mediated by STIM1 could regulate the release of inflammatory cytokines in T lymphocytes, we first analyzed the regulatory effect of STIM1 on SOCE in T cells from the trigeminal ganglion *in vivo*. Ca^2+^ content detection results showed (Figure 5A): compared with the Sham group, in the TN group, the flow of Ca^2+^ in T cells increased during the SOCE process induced by the depletion of Ca^2+^ stores in the endoplasmic reticulum while silencing STIM1 led to a decrease in Ca^2+^ flow in T cells. Experimental results from a patch clamp electrophysiology study using the chip clamp technique (Figure 5B) showed that, compared to the Sham group, the activation of ICRAC mediated by SOCE in T cells from TN group rats increased, while silencing STIM1 led to a decrease in ICRAC activation in T cells. Immunofluorescence assay results showed (Figure 5C): compared with the Sham group, the co-localization of STIM1 and ORAI1 in T cells of TN group rats increased by ~two times, while silencing STIM1 reduced the co-localization of STIM1 and ORAI1 in T cells.

Next, we examined the release of inflammatory factors following the overexpression of STIM1 and treatment with YM58483 (a SOCE inhibitor). Immunohistochemistry detected the number of T cells in rats' trigeminal ganglion. The results showed (Figure 5D): compared with the Sham group, the number of T cells in the trigeminal ganglion of the TN group rats increased significantly. Overexpression of STIM1 further increased the number of T cells in the trigeminal ganglion, while YM58483 reduced the number of T cells in the trigeminal ganglion. Quantitative changes in the number of T cells in the rat trigeminal ganglion were determined by flow cytometry. Results (Figure 5E) showed that compared to the Sham group, the number of T cells in the rat trigeminal ganglion was increased in the TN group. Overexpression of STIM1 further increased the number of T cells in the rat trigeminal ganglion, while YM58483 decreased the number of T cells in the trigeminal ganglion. The changes in inflammatory factors released by T cells in the trigeminal ganglia of rats in each group were detected by flow cytometry. The results showed (Figure 5F): compared with the Sham group, the expression of inflammatory factors IL-6, IL-1β, and TNF-α released by T cells in the trigeminal ganglia of rats in the TN group increased. Overexpression of STIM1 further increased the expression of IL-6, IL-1β, and TNF-α released by T cells in the trigeminal ganglia, while YM58483 decreased the expression of IL-6, IL-1β, and TNF-α released by T cells in the trigeminal ganglia. The above results indicate that excessive expression of STIM1 promotes the release of inflammatory cytokines in SD rats' T cells through mediating store-operated calcium entry (SOCE).

### STIM1-mediated SOCE in T lymphocytes exacerbates trigeminal neuralgia symptoms: *in vivo* modulation by YM58483

Finally, to further verify whether STIM1 regulates the release of inflammatory factors in T lymphocytes to promote trigeminal neuralgia through mediating SOCE *in vivo*, we conducted experiments by orally administering the SOCE inhibitor YM58483 to rats.

**Figure 6:**
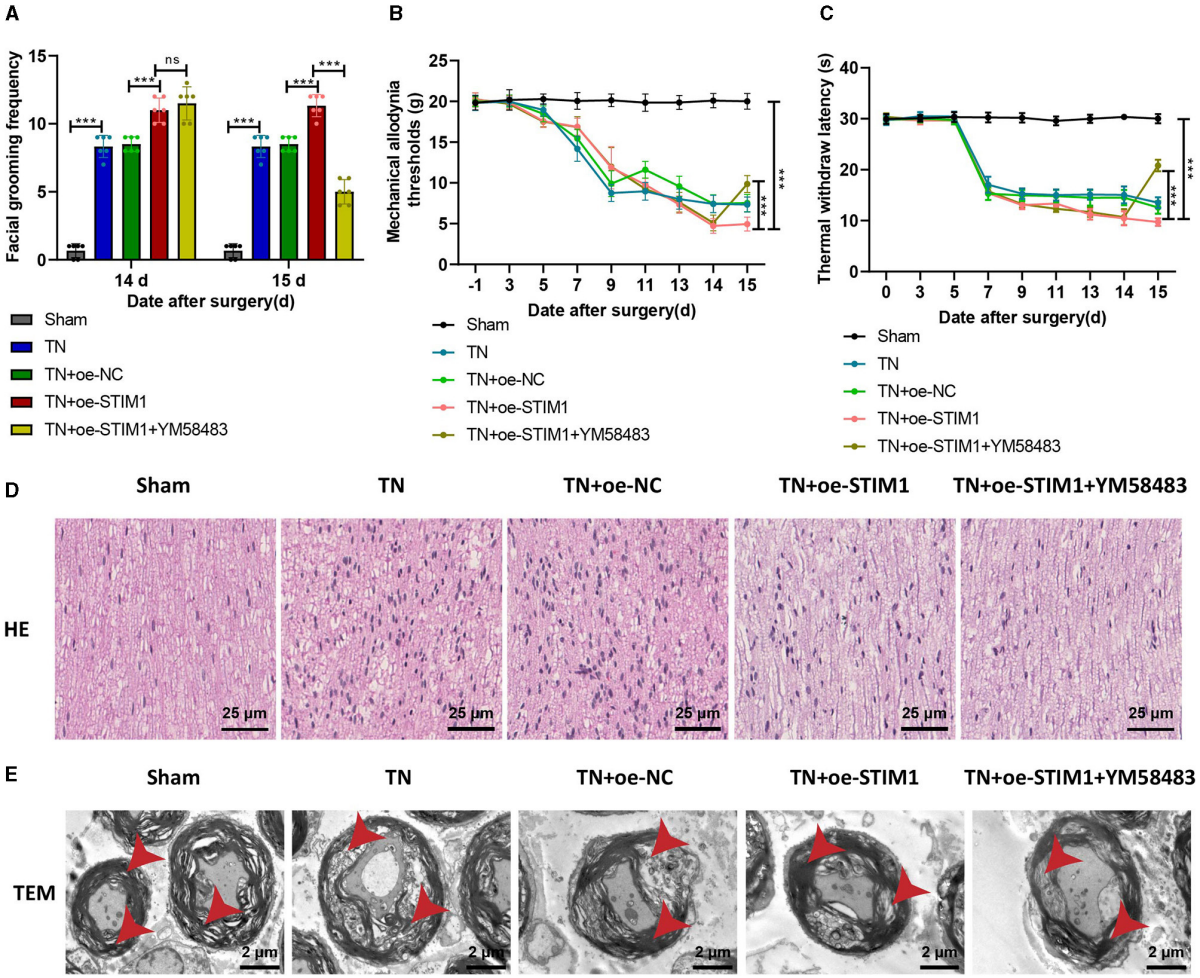
STIM1 mediates SOCE regulation of T lymphocyte inflammatory cytokine release promoting trigeminal neuralgia. **(A)** Spontaneous asymmetrical facial grooming experiment to detect changes in spontaneous facial grooming behavior in each group of rats, six rats per group. **(B)** Hot plate reflex latency test to detect changes in thermal pain threshold in each group of rats, six rats per group. **(C)** Von Frey experiments to detect changes in mechanical pain threshold in each group of rats, six rats per group. **(D)** HE staining to observe pathological changes in the trigeminal ganglion of each group of rats ( × 400), six rats per group. **(E)** Microscopic structural changes in the trigeminal ganglion of each group of rats ( × 5,000), six rats per group. Ns indicates no difference between the two groups (*P* > 0.05), ***P < 0.001.

The results indicate (Figure 6A): on day 14, the TN group rats exhibited increased spontaneous facial grooming behavior compared to the Sham group. Compared with the TN + oe-NC group, the TN + oe-STIM1 group and TN + oe-STIM1 + YM58483 group showed increased spontaneous facial grooming behavior. On the 15th day, there was no change in the behavior of the Sham group, TN group, TN + oe-NC group, and TN + oe-STIM1 group. Compared with the TN + oe-STIM1 group, the TN + oe-STIM1 + YM58483 group showed a reduction in spontaneous facial grooming behavior in rats.

The detection results of mechanical pain threshold and thermal pain threshold (Figures 6B, C): Compared with the Sham group, the mechanical pain threshold and thermal pain threshold of rats in the TN group were reduced on the right side of the face. Compared with the TN + oe-NC group, the suitable facial mechanical and heat pain threshold was decreased in the TN + oe-STIM1 group. Compared with the TN+ o e-STIM1 group, the mechanical and thermal pain threshold of the rats in the TN + oe-STIM1 + YM58483 group were elevated.

The staining results of rat HE (Figure 6D) showed that the neural fibers and Schwann cells in the Sham group were arranged neatly. The arrangement of nerve fibers in the TN group is slightly disordered, with Schwann cell proliferation, axonal swelling, lymphocyte infiltration, and demyelination changes. Compared to the TN group, the neuro fiber arrangement in the TN + oe-STIM1 group is more disordered, with Schwann cell hyperplasia, axonal swelling, lymphocyte infiltration, and demyelination alterations. The neural fibers of the TN + oe-STIM1 + YM58483 group are arranged neatly, and the degree of neural fiber degeneration is improved to varying degrees, with a reduction in inflammatory reactions.

The electron microscopy results (Figure 6E) showed that the trigeminal ganglion nerve fibers in the Sham group of rats had regular axonal morphology, with uniformly dense myelin sheath layers, clear structure, and generally consistent thickness, appearing round or oval. The cross-sectional area of the nerve fibers in the trigeminal ganglion of the TN group rats was increased, with thickened myelin sheaths, loose lamellae, disorganized structure, and local discontinuity of the myelin sheaths. The cross-sectional area of the nerve fibers in the trigeminal ganglion in the TN + oe-STIM1 group was increased, accompanied by thickened myelin sheaths, loose lamellar structure, and disrupted organization. Localized breaks in the myelin sheath were also observed. The organization structure of the TN + oe-STIM1 + YM58483 group has been improved.

In conclusion, the experimental results indicate that STIM1 promotes trigeminal neuralgia by mediating store-operated calcium entry to regulate the release of inflammatory factors in T lymphocytes.

## Discussion

In our study, bioinformatics analysis revealed the upregulation of the STIM1 gene in patients with trigeminal neuralgia, indicating its role in the occurrence and development of trigeminal neuralgia. This finding is consistent with previous research results, confirming the critical role of the STIM1 gene in pain disorders (Gang et al., 2022; Wang et al., 2023). However, this study, for the first time, explicitly associates the role of STIM1 with the pathological processes of trigeminal neuralgia, which is of importance for our understanding of the pathogenesis of trigeminal neuralgia.

Trigeminal neuralgia is a multifactorial pain disorder involving neuronal hyperexcitability, neuroinflammation, and abnormal neuronal plasticity following nerve damage (Dong et al., 2023). Our experimental results indicate that compared to TN (trigeminal neuralgia) rats, the TN + shRNA STIM1 group showed partial relief in mechanical allodynia and thermal hyperalgesia latency. This may involve a combination of various factors and mechanisms. Furthermore, despite demonstrating the critical role of STIM1 in regulating calcium ion channels, it does not exclude the presence of other calcium ion channels and signaling pathways related to pain transmission.

In this study, we further elucidated the mechanism of STIM1 regulation of the store-operated calcium entry (SOCE) pathway through Ca^2+^ content measurement, patch-clamp electrophysiology, and co-localization experiments of STIM1 and ORAI1 (Shrestha et al., 2020). Before, this mechanism was mentioned in various diseases, such as tumors and immune disorders (Zhou et al., 2022). However, this study is the first to confirm this mechanism in the pathogenesis of trigeminal neuralgia, thereby further enriching the functional research of the SOCE pathway in neurological disorders (Zhong et al., 2022). The results of this study demonstrate that STIM1 mediates store-operated calcium entry (SOCE) to promote the release of inflammatory cytokines in T lymphocytes, a novel finding that has not been reported in previous literature. This discovery reveals one of the pathological mechanisms of trigeminal neuralgia, namely that STIM1, by regulating SOCE, further affects the release of inflammatory factors in T lymphocytes, thereby influencing the occurrence of neuralgia (Zhao et al., 2020).

Our experimental results indicate that STIM1 promotes the release of inflammatory factors in T lymphocytes by mediating SOCE, thereby influencing the occurrence of trigeminal neuralgia. This result is consistent with previous studies on the role of the STIM1 gene in other neurological disorders, highlighting the role of STIM1 in neurological diseases. However, this study is the first to reveal that STIM1 affects the release of inflammatory factors in T lymphocytes, thus impacting the occurrence of trigeminal neuralgia and providing new insights for its treatment (Murphy et al., 2020).

Considering the role of STIM1 in regulating the SOCE pathway and the release of inflammatory cytokines in T lymphocytes, as well as its central position in the pathogenesis of trigeminal neuralgia, we believe that STIM1 is a novel target for the treatment of trigeminal Although drug development for STIM1 is still in the early stages, according to our research, further drug screening and efficacy evaluation targeting STIM1 could be carried out, providing new strategies for the treatment of trigeminal neuralgia.

**Figure 7:**
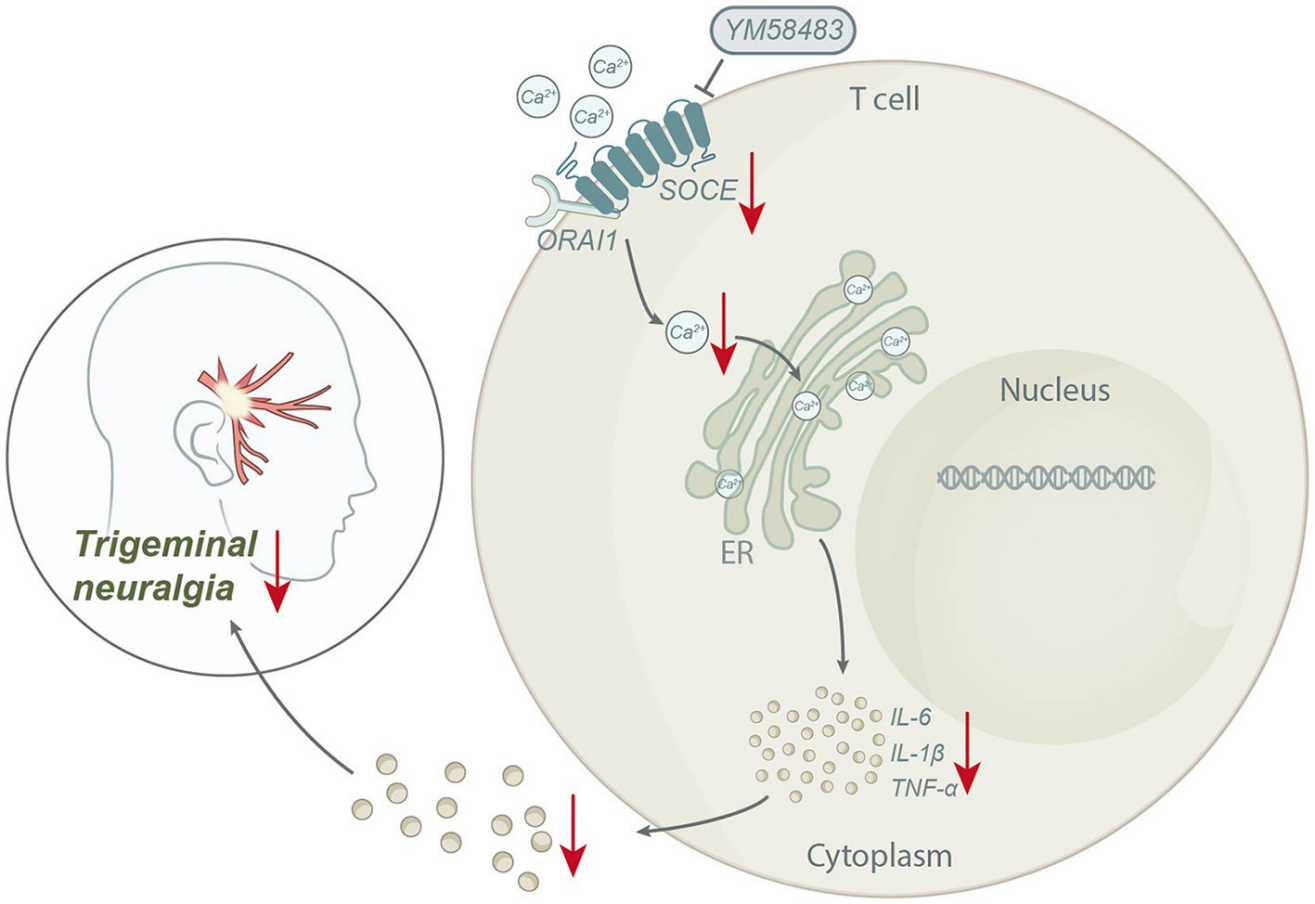
Schematic diagram of the molecular mechanism by which STIM1 mediates SOCE regulation of T lymphocyte inflammatory cytokine release to promote trigeminal neuralgia.

Based on the above results, we could conclude that STIM1 mediates SOCE in regulating T lymphocyte inflammatory cytokine release, promoting trigeminal neuralgia (Figure 7). The scientific value of this study lies in deepening our understanding of the pathogenesis of trigeminal neuralgia. By analyzing the gene expression differences of patients with trigeminal neuralgia using bioinformatics tools, we discovered the high expression of the STIM1 gene in such patients, suggesting its potential key role in the occurrence and progression of the disease. In addition, we have also demonstrated that STIM1 plays a vital role in the development of trigeminal neuralgia by regulating the calcium store-operated calcium entry (SOCE) pathway and promoting the release of inflammatory cytokines in T lymphocytes. This study provides us with a further understanding of the molecular mechanisms of trigeminal neuralgia and offers an essential theoretical basis for developing new treatment strategies.

The clinical value of this study lies in providing new possibilities for treating trigeminal neuralgia. We confirm that the STIM1 gene promotes the occurrence of trigeminal neuralgia by regulating the SOCE pathway and releasing inflammatory factors in T lymphocytes. Therefore, drugs targeting STIM1 or the SOCE pathway may become a new approach for treating trigeminal neuralgia (Zhao et al., 2020). While further clinical trials are needed to validate this, it offers new hope for patients suffering from trigeminal neuralgia.

However, this study also has some limitations. Firstly, this study was primarily conducted in cell and animal models, and further clinical research is required to validate our findings. Secondly, although we have found that STIM1 plays a crucial role in developing trigeminal neuralgia, we still need a deeper understanding of its specific mechanism of action. In addition, although we have found that STIM1 promotes the release of inflammatory cytokines in T lymphocytes by regulating the SOCE pathway, the regulatory network of the SOCE pathway is complex, and other genes and signaling pathways may also be involved, which requires further study (Zhao et al., 2020).

Looking ahead, we will continue to investigate the specific mechanism of the role of STIM1 in trigeminal neuralgia and explore its potential as a therapeutic target for trigeminal neuralgia. We will continue to study the regulatory network of the SOCE pathway to explore other potential therapeutic targets. We hope that our research could bring new treatment hope for patients suffering from trigeminal neuralgia.

## Data availability statement

The original contributions presented in the study are included in the article/Supplementary material, further inquiries can be directed to the corresponding authors. Original datasets from this study can be found here: https://figshare.com/articles/dataset/original_data_xlsx/25965424.

## Ethics statement

The animal study was approved by Animal Ethics Committee of First Affiliated Hospital of Heilongjiang University of Chinese Medicine (approved number: 2023122950). The study was conducted in accordance with the local legislation and institutional requirements.

## Author contributions

GC: Conceptualization, Data curation, Formal analysis, Funding acquisition, Writing – original draft. YZ: Data curation, Formal analysis, Investigation, Resources, Writing – review & editing. FS: Investigation, Methodology, Project administration, Resources, Writing – original draft. QZ: Software, Supervision, Validation, Visualization, Writing – review & editing.
