# Supplementary material for: Novel insights into STIM1's role in store-operated calcium entry and its implications for T-cell mediated inflammation in trigeminal neuralgia
Source: Front Mol Neurosci. 2024 Jun 19;17:1391189. doi: 10.3389/fnmol.2024.1391189 (PMC11221526; doi:10.3389/fnmol.2024.1391189)
Supplement: Supplementary file 1 [file Table_1.DOCX]

**Table S1. shRNA sequence information**

| Name | shRNA sequence |
| --- | --- |
| shRNA-NC | Sense 5’-UAAGGCUAUGAAGAGAUAC-3’ |
|  | Antisense 5’-GUAUCUCUUCAUAGCCUUA-3’ |
| shRNA-STIM1-1 (rat) | Sense 5’-CCTTGGTCCTTCATCATTATT-3’ |
|  | Antisense 5’-AATAATGATGAAGGACCAAGG-3’ |
| shRNA-STIM1-2 (rat) | Sense 5’-CTTGGTCCTTCATCATTATTT-3’ |
|  | Antisense 5’-AAATAATGATGAAGGACCAAG-3’ |
| shRNA-STIM1-3 (rat) | Sense 5’-TTGGTCCTTCATCATTATTTA-3’ |
|  | Antisense 5’-TAAATAATGATGAAGGACCAA-3’ |
